# Genetics of hereditary forms of primary hyperparathyroidism

**DOI:** 10.1007/s42000-023-00508-9

**Published:** 2023-12-01

**Authors:** Katherine A. English, Kate E. Lines, Rajesh V. Thakker

**Affiliations:** 1 OCDEM, Radcliffe Department of Medicine, Churchill Hospital, University of Oxford, Oxford, OX3 7LJ UK; 2 Oxford NIHR Biomedical Research Centre, Oxford University Hospitals Trust, Oxford, OX3 7LE UK

**Keywords:** Multiple endocrine neoplasia, Calcium-sensing receptor, PHPT, Parathyroid

## Abstract

Primary hyperparathyroidism (PHPT), a relatively common disorder characterized by hypercalcemia with raised or inappropriately normal serum parathyroid hormone (PTH) concentrations, may occur as part of a hereditary syndromic disorder or as a non-syndromic disease. The associated syndromic disorders include multiple endocrine neoplasia types 1–5 (MEN1-5) and hyperparathyroidism with jaw tumor (HPT-JT) syndromes, and the non-syndromic forms include familial hypocalciuric hypercalcemia types 1–3 (FHH1-3), familial isolated hyperparathyroidism (FIHP), and neonatal severe hyperparathyroidism (NS-HPT). Such hereditary forms may occur in > 10% of patients with PHPT, and their recognition is important for implementation of gene-specific screening protocols and investigations for other associated tumors. Syndromic PHPT tends to be multifocal and multiglandular with most patients requiring parathyroidectomy with the aim of limiting end-organ damage associated with hypercalcemia, particularly osteoporosis, nephrolithiasis, and renal failure. Some patients with non-syndromic PHPT may have mutations of the *MEN1* gene or the calcium-sensing receptor (*CASR*), whose loss of function mutations usually cause FHH1, a disorder associated with mild hypercalcemia and may follow a benign clinical course. Measurement of the urinary calcium-to-creatinine ratio clearance (UCCR) may help to distinguish patients with FHH from those with PHPT, as the majority of FHH patients have low urinary calcium excretion (UCCR < 0.01). Once genetic testing confirms a hereditary cause of PHPT, further genetic testing can be offered to the patients’ relatives and subsequent screening can be carried out in these affected family members, which prevents inappropriate testing in normal individuals.

## Introduction

Primary hyperparathyroidism (PHPT) is a relatively common disorder with an overall prevalence of 0.84–0.86% [1, 2]. PHPT is characterized by hypercalcemia, with either raised or normal (~ 80%) parathyroid hormone (PTH) concentrations [3, 4]. PHPT occurs more frequently in women than in men with a female-to-male ratio of 2–4:1 [1, 5] and is most prevalent in post-menopausal women [6]. However, in people < 50 years of age, the incidence is similar between genders [7, 8]. PHPT is usually a sporadic (i.e., non-hereditary) disease caused by a single parathyroid adenoma (~ 80%), parathyroid hyperplasia (~ 15%), multifocal disease (~ 5%), or parathyroid carcinoma (< 1%) [9]. However, such sporadic forms of PHPT most commonly occur due to somatic mutations in ~ 90% of patients [6], with the two most common genetic abnormalities being the following: loss of function (LOF) mutations in multiple endocrine neoplasia 1 (*MEN1* OMIM: 613733), which encodes for the tumor suppressor protein, menin, found in 12–35% of cases, and over-expression of cyclin D1 (encoded by *CCND1* OMIM: 168461), which is found in 20–40% of cases [9]. However, there is also increasing evidence that familial or de novo germline mutations cause PHPT as either part of a multiple tumor syndrome, e.g., MEN1, or isolated PHPT, e.g., familial isolated hyperparathyroidism (FIHP). Syndromic forms of PHPT (Table 1) include MEN1, MEN2 (formerly MEN2A) due to activating missense mutations in the rearranged during transfection protooncogene (*RET*; OMIM: 164761)), MEN4 due to LOF mutations in the cyclin-dependent kinase Inhibitor 1B (*CDKN1B*; OMIM: 600778)), MEN5 due to LOF mutations in the MYC-associated factor X (*MAX*; OMIM: 154950), and hyperparathyroidism-jaw tumor (HPT-JT) syndrome due to LOF mutations in cell division cycle 73 (*CDC73*; OMIM: 607393). Non-syndromic forms of PHPT include FIHP, familial hypocalciuric hypercalcemia (FHH), and neonatal severe primary hyperparathyroidism (NS-HPT). FIHP may be caused by germline mutations in *MEN1* [10, 11], *CDC73*, calcium-sensing receptor (*CASR;* OMIM: 601199) [12], and, as reported more recently, glial cells missing transcription factor 2 (*GCM2* OMIM: 603716) and familial hypocalciuric hypercalcemia type 1 (FHH1). Three types of FHH (FHH1-3) are recognized with FHH1 caused by LOF mutations in the *CASR*, namely, FHH2 caused by LOF mutations in the guanine nucleotide-binding protein, alpha-11 (*GNA11* OMIM: 139313) and FHH3 caused by LOF mutations in the adaptor-related protein complex 2, and sigma-1 subunit (*AP2S1* OMIM:602242; Fig. 1). This review will focus on the genetics of hereditary forms of PHPT.

**Table 1:** Syndromic forms of PHPT

Syndrome	Gene and location	Gene product	Prevalence	Associated phenotypes	% PHPT
MEN1	MEN1 11q13	Menin^a^	1–3 per 100,000	PHPT, PNETs, PA, lung carcinoids, lipomas, colagenomas, meningiomas, adrenocortical tumors, facial angiofibromas	> 90% by age 70 years
MEN2^*^ (MEN2A)	RET 10q11.2	RET^b^	13–24 per 1,000,000	MTC, pheochromocytoma, PHPT	5–15%
MEN4	CDKN1B 12p13	P27^a^	?^c^	PHPT PA Adrenal Renal Gonads	75%
MEN5	MAX 14q23.3	MAX^a^	?^d^	Paragangliomas, pheochromocytomas, PHPT, PA, PNETs	?^e^
HPT-JT	CDC73 1q31.2	Parafibromin^a^	?^f^	PHPT, ossifying fibromas of the jaw	95%

*PHPT* primary hyperparathyroidism (PHPT), *PNET* pancreatic neuroendocrine tumor, *MTC* medullary thyroid carcinoma, *PA* pituitary adenoma

^a^Loss of function

^b^Gain of function

^c^Overall, 76 cases have been reported [13, 14]

^d^Germline *MAX* mutations and pheochromocytomas in association with other endocrine tumors have been reported in 11 cases (PHPT, pituitary adenoma, and PNETs) [15–22]

^e^PHPT has been reported in four cases in patients with MEN5

^f^*CDC73* mutations are reported to account for ~ 12% of patients with hereditary PHPT [23, 24]

^*^There are three classical types of MEN2 syndrome, as follows: MEN2A (now referred to as MEN2), MEN2B (now referred to as MEN3) which is characterized by the occurrence of aggressive MTC, and pheochromocytoma in association with a Marfinoid habitus, mucosal neuromas, medullated corneal nerve fibers, and intestinal ganglioneuromas; and familial MTC, in which MTC is the sole manifestation. MEN2 has also been reported to be associated with cutaneous lichen sclerosis and Hirschsprung’s disease

**Fig. 1:**
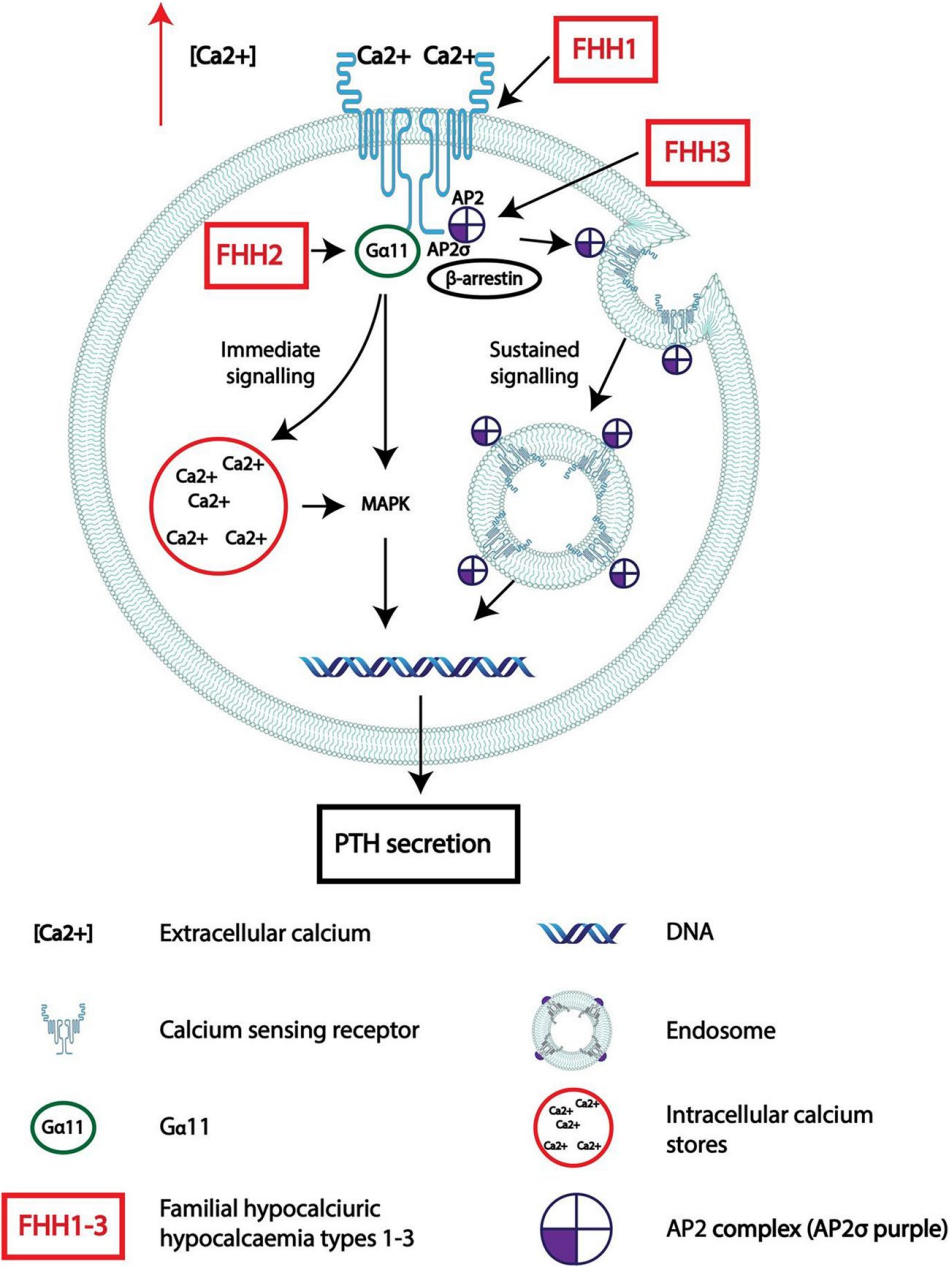
Examples of genetic changes associated with familial hypocalciuric hypercalcemia (FHH). In FHH types 1–3, inactivating mutations in the CaSR, GNA11, or AP2S1 lead to loss of function of signaling through the CaSR pathway and therefore require higher extracellular calcium concentrations (red arrow) to suppress PTH secretion. These germline genetic changes affect all parathyroid cells

## Physiology of calcium homeostasis

**Fig. 2:**
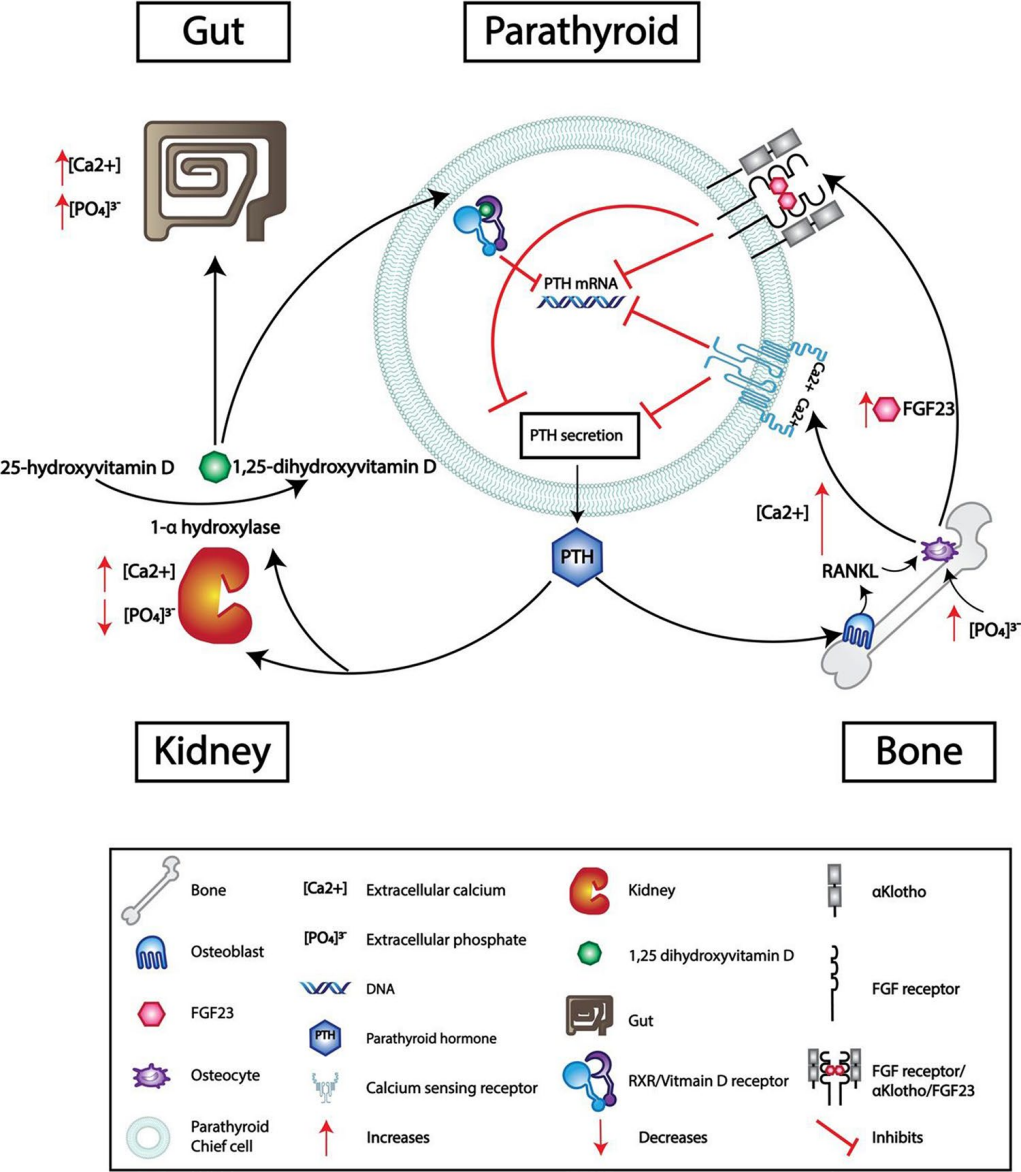
Parathyroid hormone (PTH) synthesis and/or secretion can be decreased in the chief cell of the parathyroid gland by different mechanisms, which include the following: increased extracellular calcium concentrations by activation of the calcium-sensing receptor (CaSR), activation of the fibroblast growth factor (FGF)/αKlotho receptor complex by FGF23, and activation of the retinoid X receptor (RXR)/vitamin D receptor complex by 1,25-dihydroxyvitamin D. In bone, PTH acts on the osteoblast to secrete receptor activator of nuclear factor kappa-B ligand (RANKL), which acts on the osteocytes to release calcium, thereby increasing extracellular calcium. FGF23 is released by the osteocytes in response to increased extracellular phosphate concentrations, which acts on the FGF/αKlotho receptor complex in the parathyroid gland and decreases synthesis of PTH mRNA and PTH secretion. PTH acts on the kidney to decrease the reabsorption of phosphate and increase the absorption of calcium, thereby increasing serum calcium and decreasing serum phosphate concentrations. PTH acts on 1αhydroxylase in the kidney to convert 25 hydroxyvitamin D to active 1,25-dihydroxyvitamin D, which increases both calcium and phosphate absorption and interacts with the RXR/vitamin D receptor complex in the parathyroid gland to suppress PTH mRNA

Serum calcium is maintained within a narrow range (< 0.4 mmol/L), with extracellular calcium concentrations monitored by parathyroid gland chief cells. PTH secretion from the parathyroid glands is predominantly determined by extracellular calcium concentrations [Ca2 +] by the calcium-sensing receptor (CaSR; encoded by the *CASR* gene located on chromosome 3q13.33-q21.1; Figs. 1 and 2). In addition to the direct effect of extracellular [Ca2 +], PTH is also suppressed by circulating 1,25-dihydroxyvitamin D (calcitriol or active vitamin D) concentrations which act on the vitamin D receptor (VDR), and by fibroblast growth factor 23 (FGF23) concentrations via its action on the fibroblast growth factor receptor (FGFR), in association with α-Klotho. FGF23 is released by osteocytes in response to raised extracellular phosphate and predominantly acts by increasing renal phosphate excretion and by inhibiting the renal conversion of 25-hydroxyvitamin D to 1,25-dihydroxyvitamin D (Fig. 2). The CaSR is a G-protein coupled receptor (GCPR) that is stimulated by a rise in extracellular [Ca2 +] that leads to inhibition of PTH secretion. Conversely, when the CaSR detects a decrease in extracellular [Ca2 +], signaling is reduced and PTH secretion increases. PTH works to increase serum [Ca2 +] directly at the bone and in the kidney and indirectly via the gut (by increased production of 1,25-dihydroxyvitamin D which increases gut absorption of both calcium and phosphate). At the kidney, PTH causes a decrease in phosphate absorption, predominantly by degradation of the sodium-phosphate cotransporters (NaPTs) in the proximal tubule, where up 70% of phosphate reabsorption takes place. PTH directly affects calcium reabsorption in the kidney, predominantly in the ascending limb of the renal tubule and in the distal tubule. PTH also stimulates 1-ɑ hydroxylase in the kidney, which converts 25-hydroxyvitamin D to 1,25-dihydroxyvitamin D. In bone, PTH acts on osteoblasts which release receptor activator of nuclear factor kappa-B ligand (RANKL) which, in turn, acts on osteoclasts, leading to a release of calcium from bone and, thereby, raising serum [Ca2 +].

## Pathology of PHPT and hypercalcemic disorders

The current WHO 2022 classification of parathyroid tumors aims to pathologically distinguish parathyroid disease secondary to germline mutations, i.e., multiglandular multiple parathyroid adenomas as seen with syndromic forms of PHPT (e.g., MEN1 due to *MEN1* mutations) or parathyroid carcinoma due to *CDC73* mutations, from that of parathyroid hyperplasia, usually seen in patients with secondary hyperparathyroidism (e.g., chronic renal failure) [25]. The syndromic forms of PHPT are due to LOF of tumor suppressor genes, e.g., *MEN1*, *CDKN1B*, and *CDC73*, with patients harboring germline *CDC73* mutations having a higher occurrence of parathyroid carcinomas [23, 26] or increased oncogenic signaling (e.g., activating *RET* mutations; Table 1). Some inherited non-syndromic forms of PHPT may be associated with *MEN1* [10, 11], *CASR*, e.g., FHH1, and neonatal severe hyperparathyroidism (NS-HPT) [12, 27]*,* or *CDC73* mutations [26, 28, 29]. However, it is important to note that the majority of LOF mutations of the CaSR and its signaling pathway components, the G-protein alpha 11 subunit (Gα11, encoded by *GNA11*) and the adaptor-protein 2 sigma subunit (AP2σ encoded by *AP2S1*), result in FHH1, FHH2, and FHH3, respectively (Fig. 1). The impaired signaling via the CaSR pathway in these disorders results in a higher set-point for the CaSR, which leads to hypercalcemia in association with plasma PTH concentrations that are in the normal reference range (~ 80%) or elevated [30]. In sporadic parathyroid adenomas, overexpression of *CCDN1* has been observed in 20–40% of tumors [9] and loss of expression of the CaSR has been reported in up to 90% (36/40) of tumors [31]. Additionally, promoter methylation of the *CASR* promoter 2 was found in 45% (18/40) of tumors with increased expression of the repressive histone mark trimethylation of lysine 9 on histone 3 (H3K9me3) [31]. Biochemically, elevated serum [Ca2 +] and normal or elevated PTH concentrations may be indistinguishable between the different causes of hereditary PHPT and sporadic PHPT [3, 27, 32].

## Syndromic forms of PHPT

### Multiple endocrine neoplasia 1

Multiple endocrine neoplasia 1 (MEN1) is an autosomal dominant hereditary multiple endocrine neoplasia syndrome due to a germline heterozygous LOF mutations in the *MEN1* gene, located on chromosome 11q13.1, which encodes for the 610 amino acid tumor suppressor menin. Multifocal tumors develop in endocrine glands after a second hit to the remaining functional *MEN1* allele, consistent with Knudson’s two-hit hypothesis. MEN1 is characterized by tumors in the parathyroids, pituitary, and pancreas, although other tumors can be found, including adrenal cortical adenomas and bronchopulmonary and thymic tumors [3]. The prevalence of MEN1 is reported to be between 1 and 3 per 100,000 [33]. PHPT is the most common endocrine disorder, with ~ 75% penetrance by the age of 50 years and > 90% by 70 years in MEN1 patients [34, 35]. MEN1 accounts for the majority of people presenting with PHPT due to a hereditary cause, which is in part due to MEN1 being the most common hereditary disorder associated with PHPT and is highly penetrant. More than 1500 individual MEN1 mutations have been reported in patients with MEN1 syndrome, with the majority resulting in protein-truncating variants with no clear correlation between genotype and phenotype [10, 36]. PHPT is characterized by multiple clonal parathyroid tumors that previously were classified histologically as parathyroid hyperplasia, a term which has subsequently changed with the latest WHO guidelines [25]. Menin is a scaffold protein and plays an integral role in epigenetic regulation: for example, it is required for the formation of the active histone mark trimethylation of lysine 4 on histone 3 (H3K4me3). One study reported that four parathyroid adenomas associated with MEN1 syndrome showed no global change in H3K4me3 levels by immunohistochemistry when compared to two normal parathyroid tissue samples and seven sporadic parathyroid adenomas [37]. In another study of parathyroid tissue, menin loss was reported to be associated with increased DNA methylation, a DNA mark associated with transcriptional repression [38] and 12 human MEN1-associated and one sporadic parathyroid adenoma with a L338P missense *MEN1* mutation were found to have increased global DNA methylation when compared to twelve sporadic parathyroid adenomas with no *MEN1* gene mutations and nine normal parathyroid tissue samples [38]. Finally, menin loss in seven separate parathyroid adenomas from patients with MEN1 has been reported to be associated with a reduction in the expression of the VDR when compared to both sporadic adenomas (*n* = 12) and normal parathyroid tissue (*n* = 6) [37].

### Mutation-negative MEN1

It is estimated that 10–30% of patients with a MEN1-like phenotype have no genetic mutation found in the *MEN1* gene, and these patients present with endocrine neoplasms at a later age and have a similar life expectancy to that of the general population [39]. *CDKN1B* mutations (MEN4) have been reported in ~ 1.5% of patients with mutation-negative MEN1, thereby being reclassified as having MEN4 syndrome. Germline mutations in other CDKIs have been found in 0.5–1% of patients, including *CDKN1A* (p21), *CDKN2C* (p18), and *CDKN2B* (p15) [40]. Genetic analysis for *MEN1* mutations usually involves sequencing the coding region of *MEN1* (exons 2–10); however, mutations involving the promoter region (for example, a 596 bp deletion in the *MEN1* 5′UTR) have been reported in a MEN1 kindred with no *MEN1* mutation in the coding region [41]. The significance of this deletion was tested in vivo, which reported ~ 80% reduction in MEN1 mRNA and ~ 80% reduction in menin protein expression [41]. Other causes of mutation-negative MEN1 syndrome may include the chance co-occurrence of two endocrine tumors without an underlying germline predisposition syndrome or a germline mutation in a gene not commonly screened for as part of a MEN1 panel (e.g., aryl hydrocarbon receptor-interacting protein (*AIP* OMIM: 605,555) mutations in familial isolated pituitary adenoma) with the co-occurrence of sporadic PHPT [42].

### Multiple endocrine neoplasia type 2

Multiple endocrine neoplasia type 2 (MEN2, previously MEN2A) is due to activating missense mutations in the *RET* proto-oncogene, located on chromosome 10q11.21. RET encodes a 1114 amino acid receptor tyrosine kinase which is associated with cell differentiation and proliferation. MEN2 is characterized by the occurrence of medullary thyroid carcinoma (MTC), pheochromocytomas, and PHPT. The prevalence of MEN2 is 13–24 per 1,000,000 [43]. MEN2 is more common than MEN3 (previously MEN2B; 95 vs 5%) which is not associated with PHPT [43]. MEN2 may be further classified into four subtypes, namely, classical MEN2 (MTC, pheochromocytoma and PHPT), MEN2 with cutaneous lichen sclerosis, MEN2 with Hirshsprung’s disease, or familial MTC with no other phenotype. The prevalence of PHPT in MEN2 ranges between 5 and 15%. Approximately 95% of MEN2 cases are due to activating mutations at amino acid residues 609, 611, 618, 620, and 634 (all cysteine residues), with the majority (~ 87%) at codon 634 [43, 44]. A genotype–phenotype correlation is reported with patients presenting with MEN2, in which mutations at codon 634, in particular C634R, have the highest penetrance of PHPT [45, 46].

### Multiple endocrine neoplasia type 4

Multiple endocrine neoplasia type 4 (MEN4) is characterized by germline mutations in *CDKN1B,* located on chromosome 12p13.1. *CDKN1B* transcription requires the active histone mark H3K4me3 which is maintained by a functioning menin. *CDKN1B* encodes for the 196 amino acid, p27^kip1^ or p27, a nuclear protein which is involved in cell cycle regulation and inhibits cycle progression at G1. Tumors associated with MEN4 include PHPT (75%), pituitary adenomas (44%), pancreatic neuroendocrine tumors (PNETs), papillary thyroid cancer, and renal, thymic, and reproductive organ tumors [47, 48]. MEN4 (previously termed MENX) was initially discovered in a rat [49], which developed highly penetrant multiple neuroendocrine tumors within the first year of life, with the causative gene (*CDKN1B*) discovered a few years later [50]. A recent case series and comprehensive literature review of MEN4 reported a total of 32 unique *CDKN1B* variants associated with MEN4 (with six located in the 5′UTR) from 22 studies [13]. Since then, a further two unique *CDKN1B* variants have been reported in association with familial PHPT [14]. The overall prevalence of PHPT in the entire cohort was ~ 42%, with 53.2% diagnosed with PTHP by the age of 60 years [13].

### Other genes associated with syndromic PHPT

#### Multiple endocrine neoplasia type 5 and MAX mutations

MYC-associated protein X (MAX) is a 160 amino acid protein encoded by the *MAX* gene, located on chromosome 14q23.3. The MAX protein typically forms a heterodimer with the MYC family of proteins and is involved in cellular proliferation [51]. Heterozygous LOF *MAX* mutations, which cause hereditary paraganglioma-pheochromocytoma syndrome, have also been reported with other endocrine and non-endocrine tumors [15, 52]. Multiple endocrine tumors have been associated with germline LOF *MAX* mutations, and these including pituitary adenomas and PNETs [15–19, 52]; therefore, it has been suggested that germline LOF *MAX* mutations have been suggested may cause multiple endocrine neoplasia type 5 (MEN5) [16]. There have been four reported cases of *MAX* mutations in association with PHPT [15–17, 20]. Given the rare number of case reports of PHPT in association with *MAX* mutations, further study is required to determine the role of *MAX* mutations in syndromic PHPT [53].

#### Hyperparathyroidism-jaw tumor syndrome

Cell division cycle 73 (*CDC73*; previously hyperparathyroidism type 2 (*HRPT2*)) is located on chromosome 1q31.2 and encodes a 531 protein, parafibromin; it was initially discovered in 26 affected kindreds with hyperparathyroidism-jaw tumor syndrome (HPT-JT) [54]. HPT-JT is characterized by PHPT in up to 95% of patients and ossifying fibromas in the jaw in 25–50% [28, 54–56]. HPT-JT is also associated with renal tumors including hamartomas, Wilm’s tumors, and uterine tumors [55]. Importantly, parathyroid carcinoma is over-represented in patients with *CDC73* germline or somatic mutations, which suggests that parafibromin plays an important tumor suppressor role in the parathyroid gland [23, 26]. Parafibromin is a nuclear protein with both tumor-suppressive and oncogenic properties. Parafibromin can exert its antiproliferative effect by interacting with nuclear beta-catenin by polymerase associated factor 1 (PAF1) complex [57, 58] and by decreasing the expression of cyclin D1 [59, 60], for example, by H3K9 methylation at *CCND1* and by suppression of the c-myc proto-oncogene [60, 61]. Loss of function of *CDC73* is usually by protein-truncating variants seen in up to 80% of germline variants causing HPT-JT [28]. In a cohort of 68 patients from 29 kindreds with HPT-JT, 85% presented with PHPT as their initial manifestation, with a median age of 26 years (interquartile range: 20–35 years) [23]. Of the patients with PHPT, 65% had parathyroid adenomas and 31% had features of parathyroid carcinoma [23]. PHPT is typically due to a single parathyroid adenoma, although multiple parathyroid tumors have also been reported [62, 63]. *CDC73* mutations are also seen in FIHP [62].

## Non-syndromic forms of PHPT and hypercalcemia

### Familial isolated hyperparathyroidism

Familial isolated hyperparathyroidism (FIHP) may be due to incomplete penetrance of mutations causing syndromic PHPTs, as the genes found in FIHP overlap, for example, *MEN1*, *CDC73*, or *CASR* [3, 12, 26, 28, 29, 64]. However, there is no genotype–phenotype correlation between patients with these LOF mutations and FIHP [6, 53]. Recently, activating mutations in *GCM2* located on chromosome 6p24.2, which encodes the 506 amino acid transcription factor GCMb, have been reported in 18% of patients with FIHP [65], with specific variants enriched among different ethnic backgrounds [66]. GCMb is important for parathyroid gland development, as evidenced by *Gcm2* knockout mice developing hypoparathyroidism [67] and LOF *GCM2* mutations causing familial isolated hypoparathyroidism [68]. *GCM2* activating mutations have been reported to be enriched in patients with both FIHP and sporadic PHPT; however, the penetrance appears to be low and further studies are needed [69, 70].

### Familial hypocalciuric hypercalcemia

Familial hypocalciuric hypercalcemia (FHH) is a relatively common disorder with an estimated prevalence of 74 per 100,000 [71]. FHH is caused by inactivating mutations in either *CASR* (FHH1), *GNA11* (FHH2) [72], or *AP2S1* (FHH3) [73]. FHH patients require a higher extracellular [Ca2 +] to activate the CaSR pathway, mobilize intracellular calcium, and activate mitogen-activated protein kinases (Fig. 1) [74, 75]. Therefore, patients with FHH tend to have a higher serum [Ca2 +] and normal or elevated PTH concentrations. *CASR* LOF mutations lead to a decreased ability of the kidney to excrete calcium relative to serum [Ca2 +], which results in relative hypocalciuria [76]. FHH1 is the most common form, accounting for ~ 65% of cases and, depending on the location and amino acid change in the *CASR* gene, determines the degree of loss of function and the severity of hypercalcemia [12]. There are > 230 different *CASR* variants that have been reported to be associated with FHH1. FHH2 is due to LOF mutations in *GNA11* located on chromosome 19p13.3 which encodes for Gα11. Gα11 binds to the intracytoplasmic tail of the CaSR and is responsible for signal transduction [77]. Only four variants in *GNA11* have been reported in FHH2 (T54M [78], namely, L135Q [72], I200del [72], and F220S) [79]. Pathogenic variants in *GNA11* causing FHH2 are rare and occur in < 1% of individuals undergoing genetic testing for FHH [80]. FHH3 is due to LOF mutations in the *AP2S1* gene found on chromosome 19q13.32 and encodes for the protein AP2σ. FHH3 causes < 10% of cases of FHH [73]. AP2σ is integral for CaSR endocytosis via clathrin-mediated pits and for receptor trafficking and CaSR signaling potentiation (Fig. 1) [77]. The prevalence of FHH3 has been reported at ~ 7.8 per 100,000, and mutations causing FHH3 most commonly involve the R15 residue [81]. Classical teaching describes FHH as a benign condition that needs to be distinguished from PHPT to prevent FHH patients from undergoing inappropriate parathyroidectomy [82]. One of the main distinguishing features of FHH compared to PHPT is that of low urinary calcium excretion (UCCR of < 0.01) and is seen in ~ 80% of patients with FHH type 1 and in < 20% of patients with PHPT. Other clues indicating FHH include a personal or family history of recurrence of hypercalcemia post-parathyroidectomy. However, FHH is not always a benign condition and has been associated with renal calculi, osteoporosis, and pancreatitis [12]. For patients with signs and/or symptoms suggestive of symptomatic hypercalcemia, case reports have shown efficacy with the use of cinacalcet [83]. Cinacalcet is a calcimimetic that is able to stimulate the CaSR, leading to a decrease in serum [Ca2 +] [79, 84].

### Neonatal severe hyperparathyroidism

Neonatal severe hyperparathyroidism (NS-HPT) is caused by either homozygous or compound heterozygous LOF *CASR* mutations. NS-HPT may also occur in a child with a paternally inherited (or de novo) heterozygous *CASR* mutation, born to a normocalcemic mother. NS-HPT usually presents within the first 6 months of life with life-threatening hypercalcemia, skeletal demineralization, bony deformities, fractures, constipation, dehydration, and failure to thrive. Patients with NS-HPT usually require urgent parathyroidectomy [3, 27]. There have been 12 case reports of successful treatment of NS-HPT with cinacalcet (with genetically confirmed *CASR* mutations), and three cases reported a lack of response to cinacalcet. Four of these 12 patients had a heterozygous *CASR* mutation R185Q (one inherited and three de novo) [85–87]; seven with inherited homozygous mutations, including R69H, G613E, and Y789fs [88–92]; one with a compound heterozygous mutation, C582Y and P682L [93]; and a homozygous donor splice site mutation in intron 5 [94]. All three cases reported with no response to cinacalcet were patients with homozygous *CASR* mutations at D99H, R690H, and R69H [95].

## Treatment

Special care needs to be taken in patients with syndromic PHPT as the majority present with multifocal multiglandular disease at an earlier age than sporadic PHPT; therefore, operative type, risk of recurrence, risk of post-surgical hypoparathyroidism, and age of the patient must be taken into consideration. Additionally, given the higher incidence of parathyroid carcinoma in patients with *CDC73* mutations, surveillance frequency and type of parathyroid operation will be different compared to other forms of syndromic PHPT [96]. For sporadic PHPT, patients should be considered for parathyroidectomy when there is significant risk or presence of symptomatic PHPT, for example, nephrocalcinosis or nephrolithiasis, renal failure with a creatinine clearance < 60 mL/min, hypercalciuria defined as > 250 mg/day in women and > 300 mg/day in men, minimal trauma fracture, bone mineral density by *T*-score ≤  − 2.5, or serum calcium level > 0.25 mmol/L above the upper limit of normal, or in patients who present < 50 years of age [96]. For patients in whom parathyroidectomy is contraindicated, treatment with cinacalcet may be considered: its efficacy has been reported in individuals with FHH, NS-HPT, and MEN1 [85–88, 92, 94, 97–99].

## Genetic testing for PHPT

At present, not all patients with PHPT are tested for germline mutations in genes associated with PHPT. There has been one large multicenter study that examined 1085 patients with MEN2A which found that only 10 cases presented initially with PHPT, and nine of these 10 patients were found to have synchronous MTC [100]. This suggests that the pick-up rate for diagnosing pathogenic RET mutations causing MEN2A syndrome in patients presenting only with PHPT is low and that screening for RET mutations in this scenario may not be helpful. Patients with a clinical suspicion of a hereditary form of PHPT (e.g., occurring < 30 years old, multiglandular disease, parathyroid carcinoma, first-degree relative of a known mutation carrier, or other clinical features associated with a syndromic form of PHPT) [3, 4, 96] should undergo genetic testing as this will help guide PHTP management (e.g., parathyroidectomy) and determine if screening for other tumors is required (e.g., pituitary and pancreatic neuroendocrine screening in MEN1 syndrome), while it will help determine whether family members should also be tested. For patients with a clinical suspicion of a hereditary form of PHPT, genetic testing should be undertaken; however, it is unclear if targeted genes should be tested or a PHPT panel (e.g., *MEN1*, *RET*, *CDKN1B*, *CDC73*, *CASR*, *GNA11*, and *AP2S1*) [53]. Recently, a large UK cohort study looking at 121 patients referred for genetic testing for a hereditary cause of PHPT (panel: *MEN1*, *RET*, *CDKN1A*, *CDKN1B*, *CDKN2B, CDKN2C*, *GCM2*, *CASR*, *GNA11,* and *AP2S1*) reported that 16% (19/121) of patients had a pathogenic variant in one of the following genes: 11/19 *CASR*, 6/19 *MEN1*, 1/19 *CDC73*, and 1 *AP2S1* [101].

## Conclusion

PHPT is a relatively common disorder and is associated with a genetic cause in ~ 10% of cases. Of the syndromic forms of hereditary PHPT, the MEN1 syndrome is the most common. Importantly, FHH is not as rare as originally thought and to prevent patients from undergoing inappropriate parathyroidectomy, an increased uptake of genetic testing may help with clinical decision-making. However, it is still unclear if targeted genetic testing of specific genes or testing with a global PHPT panel is the most appropriate way forward.
